# Pure Chitosan-Based Fibers Manufactured by a Wet Spinning Lab-Scale Process Using Ionic Liquids

**DOI:** 10.3390/polym14030477

**Published:** 2022-01-25

**Authors:** Irina Kuznik, Iris Kruppke, Chokri Cherif

**Affiliations:** Institute of Textile Machinery and High Performance Material Technology (ITM), Technische Universität Dresden, 01069 Dresden, Germany; iris.kruppke@tu-dresden.de (I.K.); chokri.cherif@tu-dresden.de (C.C.)

**Keywords:** chitosan, chitosan fibers, ionic liquids, fiber spinning

## Abstract

Ionic liquids offer alternative methods for the sustainable processing of natural biopolymers like chitosan. The ionic liquid 1-butyl-3-methylimidazolium acetate (BmimOAc) was successfully used for manufacturing of pure chitosan-based monofilaments by a wet spinning process at lab-scale. Commercial chitosan with 90% deacetylation degree was used for the preparation of spinning dopes with solids content of 4–8 wt.%. Rheology tests were carried out for the characterization of the viscometric properties. BmimOAc was used as a solvent and deionized water as coagulation and washing medium. Optical (scanning electron microscope (SEM), light microscope) and textile physical tests were used for the evaluation of the morphological and mechanical characteristics. The manufactured chitosan monofilaments a homogeneous structure with a diameter of ~150 μm and ~30 tex yarn count. The mechanical tests show tensile strengths of 8 cN/tex at Young’s modulus up to 4.5 GPa. This work represents a principal study for the manufacturing of pure chitosan fibers from ionic liquids and provides basic knowledge for the development of a wet spinning process.

## 1. Introduction

Chitosan is a sustainable and natural biopolymer which gives a wide range of biomedical application due to its outstanding properties like biocompatibility, biodegradability, non-toxicity, and anti-allergenic potential [1]. Chitin, which is used as raw material for chitosan production, is one of the most abundant natural substances available in nature after cellulose. Crustacean waste from the seafood industries appears as the largest industrial source of chitin. The annual worldwide production of crustacean shells amounts to 1.2 million tons, and approximately 80,000 tons of chitin is obtained from marine by-products [2]. Chitin is an unbranched polysaccharide, which is composed of β-1,4-linked N-acetylglucosamine and has a strong structural similarity to cellulose. However, due to strong intermolecular forces, chitin has a very high stability, low swelling ability, and resistance to all common solvents [3]. Chitosan as a derivative of chitin is a linear polymer of two monomers N-acetylglucosamine and glucosamine linked by a (1→4)-ß-glycosidic bond. Due to the presence of amino groups at the C-2 positions, it has a significantly improved solubility in most dilute organic acids, such as acetic or formic acid as well as some inorganic acids like HCl or HNO_3 [4]_. Chitosan is easily synthesized by a deacetylation of the raw material to a varying extent. According to this, the degree of deacetylation (DD) indicates the percentage of N-acetylglucosamine groups removed from the molecule. Diverse definitions of chitin and chitosan exist in the literature, but most sources mention a DD of 50% as a major criterion to define the substance as chitosan [1]. Chitosan is considered as one of the most suitable candidates for biomedical applications due to its unique biological properties, such as hemostatic, bacteriostatic, anticholesteremic, anticarcinogenic, or fungistatic features [5].

Ionic liquids (IL) offer a promising alternative to the commonly used acidic solvent systems for chitosan. IL are ionic compounds of organic cations and/or anions that usually have a low melting point (<100 °C). The interest in ILs occurs due to their excellent properties, such as very high thermal stability, non-inflammability, low vapor pressure, and great dissolution properties for the most known polymers [6,7,8]. Despite their unique characteristics and application prospects, there are also limitations associated with the toxicity of ILs, as different IL families revealed to have harmful effects [9]. However, due to the good recyclability and low toxicity of selected IL, the IL-technology offers environmental and safety advantages over conventional processes, like fiber production [10,11,12].

Among many types of IL, imidazolium-based IL with different counter anions are frequently used for dissolution of biopolymers, especially cellulose, making it one of the most promising solvent for the fabrication of fiber materials [13,14,15]. Since its chemical structure and solute–solvent interaction mechanisms are similar to cellulose, chitin/chitosan can be completely dissolved in IL [16,17]. Recent scientific publications demonstrate that IL, such as 1-butyl-3-methyl-imidazolium chloride (BmimCl), 1-butyl-3-methyl-imidazolium acetate (BmimOAc), or 1-ethyl-3-methyl-imidazolium acetate (EmimOAc), are also increasingly used for the preparation of microparticles, nanofibers, gels, polymer films, and membranes from chitosan [18,19,20,21]. The amount of literature describing chitosan or chitin fiber spinning from ionic liquids is still far more limited. For example, EmimOAc [22,23], glycine chloride (GlyCl) [24], or a binary system of BmimOAc and GlyCl [25] have been used for the preparation of chitosan or chitin fibers on a laboratory scale. Many scientific studies are furthermore focused on the manufacturing of composite fibers from ionic liquids, such as cellulose/chitin [26,27], or chitin/alginate blends [28].

The textile processing of chitosan offers a very high potential for the global market. The resulting structures, like fibers, textile fabrics or 3D structures, have special advantages over non-textile structures [29,30,31,32]. Some of these advantages are, for example, good draping properties and permeability of textile constructions as well as an enlarged surface with adjustable properties. Fibers made of chitin and chitosan are useful as absorbable sutures and wound-dressing materials due to wound-healing acceleration properties. Besides medical applications, chitosan fibers have a great potential for textile, cosmetic, and environmental applications, such as wastewater treatment [33,34]. The most used spinning processes to obtain a pure chitosan fiber or a blend with other compounds with higher relevance in the literature are electrospinning and wet spinning [35,36]. Wet spinning, as a common and well-known method, involves dissolution in 1–10% (*v*/*v*) acetic acid and extrusion into an alkaline bath (NaOH) to form the fibers [37]. The application of other acids is also possible [38]. The use of aqueous solutions of mineral or organic acids affords an additional post-treatment with an alkali solution to remove acids from the chitosan fibers completely. This treatment requires a high consumption of water and energy resources. Furthermore, the use of acetic acid as a solvent causes limitations for applying bioactive agents such as peptide or protein drugs, genetic material, and anticancer drugs, as the substances may be affected by acetic acid [39,40]. Therefore, it is important to implement new, highly efficient, and environmentally friendly solvents for chitosan in order to expand the product range, especially for medical applications. The implementation of IL for chitosan fiber manufacturing provides not only substantial benefits in material and processing properties but also increases the process sustainability significantly. This work represents a principal study to investigate a spinnability of chitosan fibers from ionic liquids, which provides a basis for a sustainable wet spinning process.

## 2. Materials and Methods

### 2.1. Materials

Chitosan of various qualities, DD~90%, viscosity and molecular weight (Mw) was purchased: Hydamer 90/100 (Chitinor AS, Tromsoe, Norway), in the following referred to as CHS-01; BMP 90/20 (Bulk Medicines & Pharmaceuticals GmbH, Norderstedt, Germany), in the following referred to as CHS-02, and Chitoscience 90/100 (Heppe Medical Chitosan GmbH, Halle, Germany), in the following referred to as CHS-03. Hydamer 90/100 is a chitosan with a DD of 90%, viscosity of 100 mPas (1 wt.% chitosan in 1wt.% acetic acid at 20 °C) and Mw of 100–150 kDa. Other grades used were as indicative: chitosan BMP 92/20 with Mw = 30 kDa, HMC 90/100 with Mw = 100–250 kDa. IL 1-butyl-3-methyl-imidazolium aceate BmimOAc ≥ 95 % was purchased from Sigma-Aldrich (Sigma-Aldrich Chemie GmbH, Taufkirchen, Germany).

### 2.2. Manufacturing of the Chitosan Monofilaments

In the first step, spinning dopes with solids content of 4 wt.%, 6 wt.%, and 8 wt.% were prepared by dissolving chitosan in BmimOAc, in the following referred to as CHS-R-4, CHS-R-6, and CHS-R-8, where R stands for the chitosan used. For the preparation of spinning dopes, chitosan powder and IL were weighed separately and then mixed in a flask. First, the mixture was stirred at 400–500 rpm for about 1 h to initiate the swelling processes of chitosan. In the next step, the flask was heated at a constant temperature, which was varied between 70–80 °C depending on the used chitosan material. The spinning dope solution was stirred until chitosan particles completely dissolved with a dissolution ratio, that was subsequently investigated optically and microscopically. For the preparation of the chitosan monofilaments, a laboratory syringe pump AL-4000 (WPI, Sarasota, FL, USA) was used. The spinning dope was spun into a heated coagulation bath at a certain speed. The fiber formation and separation of the IL was carried out in deionized water as the coagulant. The fibers were washed in deionized water for about 1 h at room temperature (RT) in order to completely remove the IL from the fibers. Then, the fibers were wound up and air-dried. In this test series, winding was carried out without drawing of the fibers. The IL recycling was performed by a rotary evaporator (Hei-VAP Core, Heidolph Instruments GmbH & Co. KG, Kelheim, Germany) allowing the almost complete recycling of the IL.

### 2.3. Rheology

A Haake MARS II (Thermo Fisher Scientific, Karlsruhe, Germany) stress-controlled rheometer was used for the measurements of the solutions viscosity parameters. Cone-plate geometry (35 mm diameter, 2°, Ti) was applied for the shear measurements and the oscillatory movement. The gap was 0.100 mm for all solutions at a temperature of 65 ± 0.1 °C.

### 2.4. Morphological Examination

Scanning electron microscope (SEM) images were obtained by an ESEM QUANTA FEG 250 scanning electron microscope (FEI Company, Hillsboro, OR, USA) at 500× magnification and 2.80 kV voltage. For the diameter, determination of a light microscope Imager.M1 with a digital camera AxioCam MRc5 (Carl Zeiss, Oberkochen, Germany) was utilized. The images were evaluated with ZEN (Carl Zeiss, Oberkochen, Germany) software.

### 2.5. Infrared Spectroscopy (FTIR)

IR spectra were obtained by using the Attenuated Total Reflection method on a Nicolet 6700 FTIR spectrometer (Thermo Fisher Scientific, Waltham, MA, USA). A total of 64 scans were taken and averaged from 4000 to 600 cm^−1^ with a resolution of 4 cm^−1^ for each spectrum. Omnic^TM^ Spectra software (Thermo Fisher Scientific, Waltham, MA, USA) was applied for the evaluation.

### 2.6. Textile Physical Evaluation

The linear density, *T_t_* in dtex, of the chitosan fibers was determined by the gravimetric method according to DIN EN ISO 1973. The weight of ten fiber samples with a cut length of 100 mm was determined on a microbalance. The average linear density was calculated according to the following formula:

(1) $$T_{t}=\sum_{i=1}^{10}10000\times \frac{weight\;m\;\left[\mathrm{kg}\right]}{cutting\;length\;\left[\mathrm{mm}\right]}$$

The mechanical properties were determined in a tensile/elongation test according to DIN EN ISO 5079 on the tensile testing machine (ZwickRoel, Ulm, Germany) at standard climate conditions (20 °C, 65% relative humidity (RH)). Test samples were clamped with 20 mm clamping length. The measurements were carried out at the test speed of 20 mm/min. For the knot tensile test, a loose knot with a diameter of 4 mm was formed from the fiber sample and clamped in the middle of the test length of 20 mm. The measurements were carried out at the test speed of 2 mm/min. The preload force was set at 0.05 N. The force transducer of 5 N was applied for all measurement series. The evaluation of the measured data was conducted using the testExpert III software (ZwickRoel, Ulm, Germany).

## 3. Results and Discussion

### 3.1. Dissolution of Chitosan in IL

**Figure 1:**
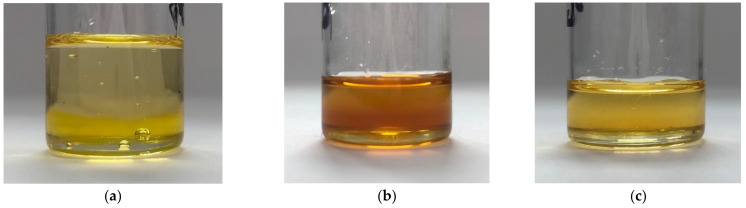
Spinning dopes of chitosan in ionic liquid 1-butyl-3-methyl-imidazolium aceate (BmimOAc) with 6 wt.% solids content: CHS-01 (**a**), CHS-02 (**b**), and CHS-03 (**c**).

When completely dissolved, the spinning solution appeared clear, transparent, and homogeneous with no traces of chitosan flakes or foreign particles. Clear, gold-colored spinning dopes were produced, with color intensity and viscosity depending on the solids concentration of chitosan (Figure 1). The viscosity of the spinning dopes increased steadily with the rise of concentration. The samples with 8 wt.% solids content showed almost a gel-like character. A relatively strong dependence of the IL dissolving power on temperature was observed. Samples with higher solids content appeared completely dissolved at 80 °C, while low concentrated samples were dissolved at lower temperatures (65–70 °C). The time required for complete dissolution was approximately 1.5–3 h, depending on the chitosan concentration.

### 3.2. Rheological Results

**Figure 2:**
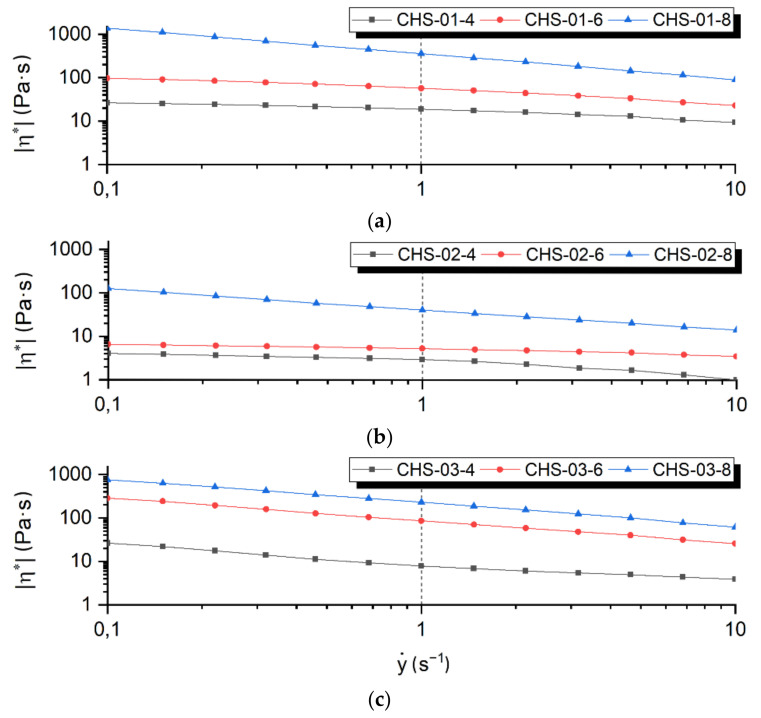
Viscosity of spinning dopes with solids contents of 4 wt.%, 6 wt.%, and 8 wt.% prepared from ionic liquid BmimOAc and chitosan CHS-01 (**a**), CHS-02 (**b**), and CHS-03 (**c**).

The results of the viscosity measurement show that all spinning dopes demonstrate a pseudoplastic behavior, which is a typical feature of polymer solutions. At the resting state or at low shear rates below 0.1 s^−1^, all samples showed high viscosities, which are reduced with increasing, shear rates (Figure 2). In the resting state, a network of intermolecular bonds is formed, which gives the spinning dope a solid character with a very high viscosity [41]. An increase of the velocity gradient causes a viscosity drop of the spinning dope due to molecular orientation in the flow direction. In addition, the viscosity shows a strong dependence on the concentration of the spinning solutions, which increases significantly with higher solids content. Shear rates of about 1 s^−1^ represent the range close to the spinning process on an industrial spinning plant [42]. At this point spinning dopes of CHS-02 show relatively low viscosities, primarily due to the low molecular mass. The viscosity of the CHS-02-4 spinning dope amounted to 3 Pa·s and increase to 5.3 Pa·s or 41 Pa·s with the rise of the solids concentration of the samples CHS-02-6 and CHS-02-8, respectively. The spinning dope from CHS-01 shows viscosity values of 18 Pa·s and 56 Pa·s of samples CHS-01-4 and CHS-01-6. Here, good spinnability into chitosan fibers was expected. By CHS-01-8 the value increases up to 358 Pa·s. A similar tendency is observed for the CHS-03 spinning solutions. The viscosities for the CHS-03-4 and CHS-02-6 samples are in the range of 10–80 Pa·s. The values increase to ~250 Pa·s by the CHS-03-8 sample.

All spinning dopes from used chitosans in at least two concentrations is successfully processed into fibers. Spinning dopes CHS-03-8 and CHS-01-8 cannot be processed into monofilaments. The limitations here were the high viscosity of the spinning dopes and the possible working range of the equipment used. Furthermore, it is impossible to produce fibers from the CHS-02-4 due to a lack of structural stability. Homogenous monofilaments with an even surface and fiber fineness of 10–45 tex is achieved from the remaining samples.

### 3.3. FTIR-Spectroscopy

**Figure 3:**
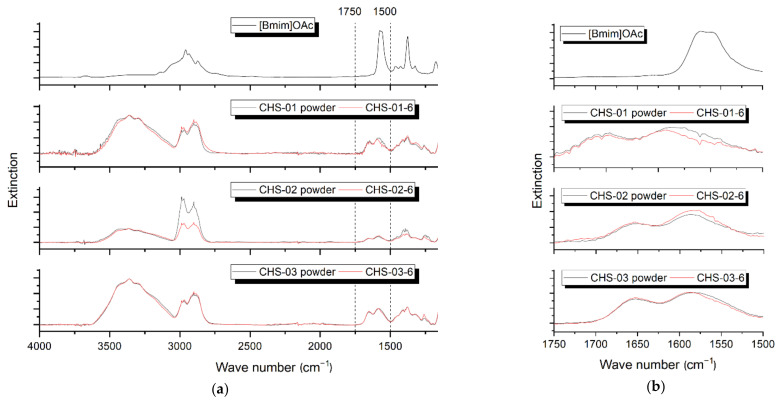
(**a**) FT-IR spectra of the ionic liquid BmimOAc, chitosan powders (CHS-01, CHS-02, CHS-03) and fibers made from the powders with BmimOAc as solvent (CHS-01-6, CHS-02-6, CHS-03-6 with 6 wt.%); (**b**) section of the wave number range of 1600–1550 cm^−1^.

The removal of the solvent BmimOAc from the chitosan monofilaments by a post-treatment with deionized water was evaluated by FTIR spectroscopy. Figure 3a presents the IR spectra of the solvent and the chitosan fibers compared to the IR spectra of the original chitosan powder. The spectra of the chitosan fibers and powders were normalized to the OH band, as BmimOAc has no signal in this range. The spectrum of BmimOAc is characterized by five significant bands: 2958 cm^−1^ υ(C-H), 1600–1550 cm^−1^ υ_as_(C=O) and 1376 cm^−1^ υ_s_(C=O), 1323 cm^−1^ δ(CH3), 1174 cm^−1^ υ(ring), and δ(NCN) [43,44,45].

In comparison, the IR spectra of all chitosan monofilament samples show the typical absorption peaks for chitosan: 3750–3000 cm^−1^ υ(OH), 2930 cm^−1^ and 2875 cm^−1^ υ(C-H), 1660 cm^−1^ υ(C=O) (amide I) and 1580 cm^−1^ δ(NH2) (amide II) [46,47], 1100–1000 cm^−1^ υ(C-O-C) [48,49]. The identical bands observed by the raw material and the fibers show that all of the examined samples contain no traces of the BmimOAc. The different intensity of a light absorption of the C-H band of the CHS-02-6 compared to the CHS-02 powder results from challenges of sample preparation due to increased brittleness of the sample.

Figure 3b shows a section of the relevant wave number range of 1750–1500 cm^−1^ with the C=O stretching. The comparison of the characteristic C=O band represents no significant difference between the signal strength of the chitosan fibers and powders. According to the measurement results, the solvent is completely removed from the chitosan fibers in the wavelength-dependent detection range of the measuring instrument. The chemical structure of chitosan is maintained within the fibers after washing in the deionized water.

### 3.4. CHS Fiber Morphology and Mechanical Properties

**Figure 4:**
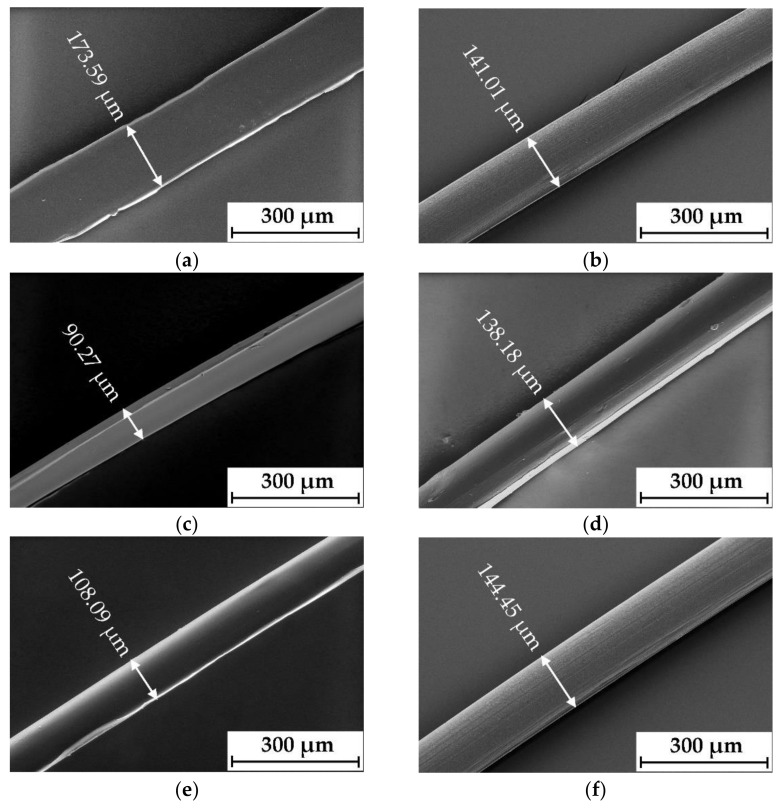
Scanning electron microscope (SEM) images of fibers made from chitosan and ionic liquid BmimOAc with different solids content: (**a**) CHS-02-4; (**b**) CHS-01-4; (**c**) CHS-03-4; (**d**) CHS-02-6; (**e**) CHS-01-6; (**f**) CHS-03-6.

The SEM and light microscopic images demonstrate a dependency of the chitosan fibers on the solid concentration. All samples with 4 wt.% show a flat cross section and diameter variation (Figure 4a–c), while the fibers with 6 wt.% chitosan have an even, round cross section with a constant diameter of 138–145 μm (Figure 4d–f) depending on the raw material. All samples show a consistent, smooth, and bright surface. The insufficient stability of the fibers with 4 wt.% can be attributed to the low solid content of the spinning solutions. With increasing chitosan concentration, the fibrillar fiber texture and associated molecular orientation increased significantly. In summary, spinning solutions with higher chitosan concentrations of 6 wt.% are more suitable for the manufacturing of chitosan fibers with very good morphological properties.

The tested chitosan monofilaments possess tenacities from 4 to 6 cN/tex and Young’s modulus of 1.5–4.5 GPa (Table 1). Due to the deficient texture, numerous surface defects, and major diameter variations, the sample CHS-02-4 is rejected for mechanical analysis. The tensile strength of the CHS-02 samples remains in the same range at ~5 cN/tex after increasing the solids content from 6 wt.% to 8 wt.% while the Young’s modulus decreases significantly from 3.2 GPa to 2.5 GPa. A high content of residual protein in the raw material represents a possible cause. In contrast, the Young’s modulus of the CHS-01 samples remains constant at ~3.5 GPa unaffected by the solids content. An increasing number of molecules and chemical bonds between them explains the growth of tensile strength with higher chitosan concentrations from 4.4 cN/tex to 6.1 cN/tex. The CHS-03-4 sample demonstrates comparable values of approx. 5.5 cN/tex and Young’s modulus of approx. 4.6 GPa already at 4 wt.% solids content. The values indicate a higher quality and purity of the raw material. The significant reduction of the values for the CHS-03-6 sample implements an irregular fiber structure due to the insufficient solubility of the raw material in IL and inhomogeneity of the spinning solution as a result.

**Figure 5:**
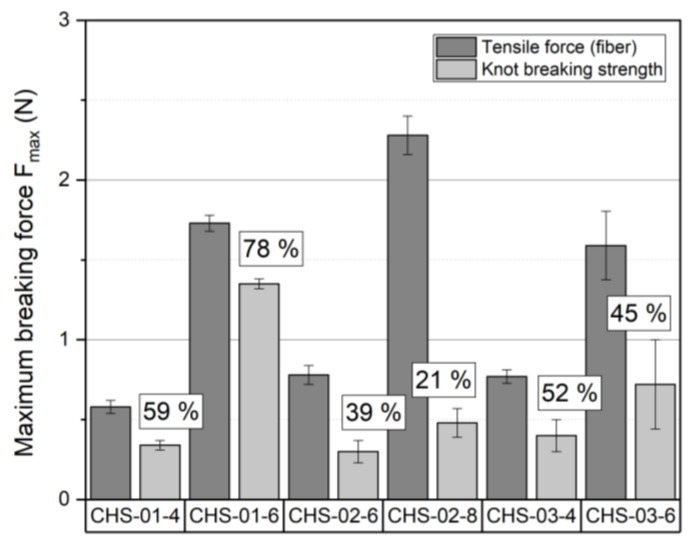
Comparison between the maximum tensile force and maximum knot breaking strength after a knot formation for the fibers from chitosan and ionic liquid BmimOAc with different solids content (knot breaking strength is additionally outlined with X% of the initial tensile force).

Knot strength represents a necessary material characteristic for different textile applications as, for example, surgical suture material. Mechanical strength and sufficient elasticity are required to form a tight knot. Especially in the case of suture material, the knot breaking strength may not fall below the maximum tensile force to prevent a material failure. The results of the knot tensile tests show that all fiber samples do not completely fulfill this requirement (Table 1). The sample CHS-01-6 demonstrates that the highest knot strength is 78% of the original tensile force. For all other samples, the knot strength decreases partially up to 20–50% of the original tensile force. The lowest values of the knot strength are observed in the fibers from the chitosan CHS-01. After knot formation, the sample with the highest tensile strength of 2.28 N has only 21.1% of the original breaking load. It is clear that the samples with the best surface and filament structure represent better results. The relatively high standard deviation provides further evidence of the relationship between the poor morphological properties and the decrease in tensile force after nodule formation. Figure 5 shows that the knot strength increases for all samples with higher chitosan concentration. It is still remarkable that the CHS-02-8 sample shows a high loss of the original tensile force despite a relatively even fiber structure. A possible explanation for this phenomenon is given by the low molecular weight and the lack of purity of the raw material. In order to investigate the full potential of the chitosan filaments as well as the effect of the impurities of the solvent and raw material on mechanical properties, the fiber drawing will be implemented in the future research. By increasing the molecular orientation through filament drawing stable structures are formed, which lead to a significant improvement of the textile physical properties.

## 4. Conclusions

Ionic liquid BmimOAc was successfully used as a solvent for the manufacturing of the pure chitosan monofilaments in a laboratory scale by wet spinning in this principal study. Commercial chitosan with a deacetylation degree of 90% was used as a raw material. Chitosan monofilaments with valuable morphological and mechanical properties were fabricated. Due to its excellent solubility, BmimOAc was used as an efficient and environmentally friendly alternative to the conventional chitosan solvents as acetic acid. The process did not require any additional chemicals for neutralization of the solvent. BmimOAc was washed out of the chitosan monofilaments with deionized water completely, as demonstrated in the FT-IR studies. With an efficient design of the wet spinning process in an industrial scale as well as further research of solvent preparation, an efficient production process can be achieved. The application of green chemistry principles represented by the use of ILs in the development of innovative biomaterial products based on chitosan fibers promotes a major sustainable utilization of this polymer, opening up new application possibilities in the biomedical field.

## Figures and Tables

**Table 1:** Mechanical properties of fibers made from pure chitosan (90% degree of deacetylation) and ionic liquid BmimOAc with different solids content.

Sample	Yarn Count (tex)	Diameter (µm)	Tensile Force (N)	Tenacity (cN/tex)	Elongation at Break (%)	Young’s Modulus (GPa)	Knot Strength (N)
CHS-01-4	13.1	irregular	0.58 ± 0.04	4.41 ± 0.29	18.20 ± 4.42	3.52 ± 0.31	0.34 ± 0.03
CHS-01-6	28.5	156 ± 2	1.73 ± 0.05	6.09 ± 0.17	33.45 ± 6.91	3.59 ± 0.23	1.35 ± 0.03
CHS-02-4	5.1	irregular	--	--	--	--	--
CHS-02-6	17.3	146 ± 5	0.78 ± 0.06	4.51 ± 0.33	14.85 ± 4.26	3.20 ± 0.21	0.30 ± 0.07
CHS-02-8	44.7	205 ± 8	2.28 ± 0.12	5.11 ± 0.27	13.19 ± 2.61	2.57 ± 0.33	0.48 ± 0.09
CHS-03-4	14.2	irregular	0.77 ± 0.04	5.44 ± 0.29	15.13 ± 5.03	4.56 ± 0.25	0.40 ± 0.10
CHS-03-6	35.3	145 ± 6	1.59 ± 0.21	4.51 ± 0.61	12.95 ± 7.30	2.86 ± 0.25	0.72 ± 0.28

## Data Availability

The data presented in this study are available on request from the corresponding author.
